# Characterization of thermostable alkaline proteases from *Bacillus infantis* SKS1 isolated from garden soil

**DOI:** 10.1371/journal.pone.0188724

**Published:** 2017-11-30

**Authors:** Sandeep Kaur Saggu, Prakash Chandra Mishra

**Affiliations:** Department of Biotechnology, Guru Nanak Dev University, Amritsar, Punjab, India; Russian Academy of Medical Sciences, RUSSIAN FEDERATION

## Abstract

Proteases are one of the largest groups of hydrolytic enzymes constituting about 60% of total worldwide sales of industrial enzymes due to their wide applications in detergent, leather, textile, food and pharmaceutical industry. Microbial proteases have been preferred over animal and plant proteases because of their fundamental features and ease in production. *Bacillus infantis* SKS1, an alkaline protease producing bacteria has been isolated from garden soil of north India and identified using morphological, biochemical and molecular methods. 16S rDNA sequence amplified using universal primers has 99% sequence identity with corresponding gene sequence of *Bacillus infantis* strain FM 34 and *Bacillus sp*. Beige. The bacterial culture and its 16S rDNA gene sequence have been deposited to Microbial Culture Collection (Pune, India) with accession number MCC 3035 and GenBank with accession number KR092197 respectively. The partially purified extract of *Bacillus infantis* SKS1 was thermostable and active in presence of Mg^2+^, acetyl acetone and laundry detergents implicating its application in industry. Production of these enzymes using this strain was maximized by optimization of various parameters including temperature, pH, media components and other growth conditions. Our results show that fructose and dextrose serve as the best carbon sources for production of these enzymes, highlighting the use of this strain for enzyme production utilizing relatively inexpensive substrates like beet molasses and corn steep liquor. Additionally, this strain showed maximum production of enzymes at 40°C similar to bacterial species used for commercial production of alkaline proteases. Characterization of alkaline proteases from this strain of *Bacillus infantis* and optimization of parameters for its production would help in understanding its industrial application and large-scale production.

## Introduction

Enzymes are biocatalysts that play a vital role in everyday life [1]. In modern world, eco-friendly enzymatic methods are replacing chemical processes. Proteases constitute about 60% of the total enzyme market making them one of the most dominant hydrolytic enzymes possessing a wide range of applications in physiological and industrial fields [2–4]. According to Business Company Research report (BCC Jan, 2017), the worldwide market for industrial enzymes was around $4.9 billion in 2015 and is supposed to reach around $6.3 billion by 2021 [5]. Proteases are the enzymes that are used in industries such as detergent, leather, waste treatment, therapeutics, diagnostics, silk degumming, silver recovery, peptide synthesis, baking and brewing [6–8]. In contrast to chemical treatments, protease based processes are specific in action and eco- friendly.

Proteases are classified as exo-peptidases (aminopeptidases and carboxypeptidases) and endo-peptidases (serine, cysteine, aspartic, metallo, threonine, glutamic endopeptidases and asparagine peptide lyases) based on position of cleavage of peptide bonds [8–10]. Based on pH, proteases can be classified as alkaline, neutral and acidic [6]. Alkaline proteases are quite important in industries as they have the capability to withstand higher pH conditions [11, 12]. Alkaline protease producing bacteria are ubiquitously found in natural [13–16] and, anthropogenic man-made environment [17]. As compared to plants and animals as a source of proteases [6, 7], it is more propitious to use microbes because of their enormous diversity, expeditious growth, requirement for limited space during cultivation and easy genetic manipulation [18, 19]. *Bacillus* is one of the most vital genera that have been used for alkaline proteases production because of their chemoorganotrophic characteristics, tremendous growth rates, secretion of extracellular enzyme into media and are safe to handle [2, 20].

Genes coding for alkaline proteases are ubiquitously present in almost all microorganisms but not every microbe can be used for commercial production of proteases. The microorganisms that are capable of producing industrially suitable proteases at economical cost are used for commercial production. Although details related to cost of industrial production or fermentation are rarely published, it is well known that fermentation economics depends on media and energy consumed during fermentation process. Recovery and purification of enzymes also add to the production cost. Therefore, there is always a need to explore new microorganisms producing novel enzymes using inexpensive fermentation processes for economical production. The aim of our study was to search a new microorganism that is capable of utilizing inexpensive carbon and nitrogen sources preferably waste products. This study deals with identification and characterization of the strain *Bacillus infantis* SKS1 and its alkaline protease. The activity of proteases produced by this strain was determined varying different parameters such as pH, temperatures, metal salts, solvents and local laundry detergents. Various physico-chemical parameters were optimized for maximum production of alkaline protease. As per our knowledge, this is the first report of alkaline protease production of *Bacillus infantis*.

## Materials and methods

### Isolation and screening of microorganism

*Bacillus infantis* SKS1 has been isolated from garden soil of Guru Nanak Dev University campus, Amritsar, Punjab, India **(**31.634 N latitude and 74.872 E longitude). Soil was collected by scraping the upper surface layer till 0–5 cm depth using 50 ml tube. Things like pebbles, rocks and leaves were removed before the soil was air-dried and used further for isolation of microbes. Screening for bacteria capable of producing alkaline protease was performed using serial dilution method on skim milk agar plates (pH 10) incubated at 37°C. Bacterial colonies forming zone of clearance on skim milk agar plates (pH 10) were isolated and purified using streaking methods. The pure cultures were stored as glycerol stocks and maintained on nutrient agar plates by sub-culturing every two weeks. The culture was submitted to Microbial Culture Collection, Pune (India) with accession number MCC 3035.

### Identification of strain SKS1

*Bacillus infantis* SKS1 was characterized using morphological, biochemical and molecular methods. Morphological characterization was done using stereomicroscope (Magnus, India) and Gram staining method using brightfield microscope (Nikon H600L, Japan). The bacterial isolate was characterized biochemically by performing carbohydrate utilization tests using HiCarbo kit (HiMedia, India) [21]. A single isolated colony of *Bacillus infantis* SKS1 was inoculated in 5 ml Brain Heart Infusion Broth (HiMedia, India) and incubated at 35–37°C for 4–6 hours until the inoculum turbidity reached 0.5 OD at 620 nm. Each well was inoculated with 50 μl of the above inoculum using surface inoculation method and incubated at 35 ± 2°C for 18–24 hours (Table 1).

**Table 1:** Results interpretation table provided with HiCarbo kit.

Test	Principle	Original colour of the medium	Positive reaction	Negative reaction
Carbohydrate utilization	Detects carbohydrate utilization	Pinkish Red / Red	Yellow	Pinkish Red / Red
ONPG	Detects β-galactosidase activity	Colourless	Yellow	Colourless
Esculin hydrolysis	Detects esculin hydrolysis	Cream	Black	Cream
Citrate utilization	Detects capability of organism to utilize citrate as a sole carbon source	Green	Blue	Green
Malonate utilization	Detects capability of organism to utilize sodium malonate as a sole carbon source	Light green	Blue	Light green

The results of carbohydrate utilization tests were interpreted according to the table provided by the manufacturer of HiCarbo kit (HiMedia, India). In case of microorganisms showing weak reaction during carbohydrate fermentation test, the reaction was recorded as ± and incubated further for 48 hours. Orange colour after 48 hours of incubation was interpreted as a negative reaction.

The ability of bacteria to produce lipase, amylase, lysine decarboxylase, indole and acetylmethylcarbinol was detected using respective media [22]. The strain was tested for antibiotic susceptibility by disc diffusion method using antibiotic octodiscs (HiMedia, India) [22]. The plates were prepared with Mueller Hinton Agar (HiMedia, India). A single colony was inoculated in 5 ml Nutrient broth and incubated at 35–37°C for 4–8 hours until the inoculum turbidity reached 0.2 OD at 600nm. The discs were applied aseptically using sterile forceps on Mueller Hinton agar plates spread with 100 μl cultures. Theses plates were incubated immediately at 35 ± 2°C and examined after 16–18 hours or longer for zones showing complete inhibition. Two types of antibiotic octodiscs, G plus 17 (HiMedia, India) and G-XIV- plus (HiMedia, India) were used for the study as per the concentration mentioned in Table 2.

**Table 2:** Concentration of antibiotics discs.

Antibiotic	Antibiotic concentration
Co-trimoxazole (COT)	25 μg
Cefepime (CPM)	30 μg
Quinupristin/Dalfopristin (RP)	15 μg
Ceftriaxone (CTR)	30 μg
Doxycycline (DO)	30 μg
Levofloxacin (LE)	5 μg
Amikacin (AK)	30 μg
Sparfloxacin (SPX)	5 μg
Penicillin-G (P)	10 units
Gentamicin (GEN)	10 μg
Augmentin (AMC)	30 μg
Ciprofloxacin (CIP)	5 μg
Erythromycin E	10 µg
Fusidic acid (FC)	10 µg
Chloramphenicol (C)	30 µg
Vancomycin (VA)	30 µg

Antibiotic discs were used for antibiotic susceptibility tests against *Bacillus infantis* SKS1.

16S rDNA sequence obtained by sequencing of PCR amplified product using a set of universal bacterial primers i.e. 8F (5’-AGAGTTTGATCCTGGCTCAG-3’) and 1492R (5’-GGTTACCTTGTTACGACTT-3’) was used for molecular characterization. 16S rDNA sequence (sequenced by First Base Laboratories Sdn Bhd, Malaysia) was deposited in GenBank having accession number KR092197. 16S rDNA sequence was used to search NCBI [23], Ribosomal database project-II [24] and EzTaxon [25] for homologous sequences. Phylogenetic study was done by neighbour-joining method using Maximum Composite Likelihood as correction factor by Mega 6 software with bootstrap value of 1000 replicates [26]. Clustal W (MEGA 6) was used for alignment. *Lentibacillus* was chosen as an outgroup being one of the closest members to the strains present in ingroup. It is a member of family Bacillaceae 2 whereas other strains belong to Bacillaceae 1.

### Protease assay

Protease activity was determined using modified Lowry method [27, 28]. Reaction mixture containing 2 ml buffered casein (pH 10) and 1 ml partial purified extract (PPE) was incubated for 20 minutes at 40°C. “Casein protein rich refined” (Sisco Research Laboratories) at 1% concentration was used for protease assay. The reaction was stopped by adding 3 ml of 10% trichloroacetic acid (Sisco Research Laboratories, India) and incubated for 10 minutes at room temperature followed by centrifugation at 10,000 rpm. 4 ml sodium carbonate (0.4M) and 500 µl Folin phenol Ciocalteau reagent (1N) were added to 1 ml supernatant followed by incubation for half an hour at room temperature and absorbance was measured at 660 nm. One unit enzyme activity was defined as the amount of enzyme that is needed to release 1µg tyrosine per ml per minute under standard assay conditions.

### Characterization of alkaline protease

Ammonium sulphate (70% ammonium sulphate) method was used for precipitation of enzyme. Pellet obtained after precipitation was resuspended in 50 mM Tris buffer (pH 8) which was further dialyzed with 50 mM Tris buffer (pH 8) and then used for PPE characterization. The protein content and protease activity was determined and specific activity was calculated as enzyme activity per mg protein.

Following buffers (0.1 M) at different pH ranging from 6 to 12 were used to determine the effect of pH on protease activity (Table 3).

**Table 3:** Buffers used for determining pH effect on enzyme activity.

Buffers (0.1 M)	pH
Potassium phosphate buffer	6
Potassium phosphate buffer	7
Potassium phosphate buffer	8
Sodium carbonate–bicarbonate buffer	9
Sodium carbonate–bicarbonate buffer	10
Sodium bicarbonate–sodium hydroxide buffer	11
Potassium chloride–sodium hydroxide buffer	12

Various buffers having pH ranging from 6 to 12 were used to study effect of pH on protease activity.

The effect of temperature on protease activity was analysed by incubating reaction mixture containing partially purified extract (PPE) and buffered casein for an at 20°C, 30°C,40°C, 50°C, 60°C, 70°C, 80°C, 90°C and 100°C. The PPE was pre-incubated for an hour at 20°C, 30°C, 40°C, 50°C, 60°C, 70°C, 80°C, 90°C and 100°C before adding to reaction mixture to check thermostability of enzyme. The reaction mixture containing PPE was incubated for an hour with salts of various metal ions (Mg^2+^, K^+^, Mn^2+^, Li^+^, Fe^3+^, Na^+^, Zn^2+^, Ba^2+^ and Co^2+^) at 0.25% to examine catalytic effect of metal ions on enzyme activity taking PPE alone as control. The reaction mixture was incubated with various solvents such as acetone, acetyl acetone, methanol, isopropanol, ethyl acetate, dodecane, hexadecane, carbon tetrachloride, N, N- dimethyl formaldehyde, isobutanol, isoamyl alcohol, chloroform, petroleum ether, formamide, toluene and ethylene glycol at 0.25% concentration at 37°C to analyse the activity of PPE in presence of various solvents taking PPE alone as control. Five laundry detergents (solid) such as Ariel (Procter and Gamble), Fena (Gautum Co-operative Industrial Society Limited), Ghari (Rohit Surfactant Private Limited), Surf Excel Matic (Hindustan Lever Limited) and Henko (Henkel Spic India Limited) at 7 mg/ml concentration were used to study the compatibility of PPE with laundry detergents. Endogenous proteases present in laundry detergents (solid) were inactivated by incubating them at 100ºC for an hour and PPE alone was taken as control. Protease assay was performed with inactivated detergents (controls) but no activity was detected in detergents with inactivated proteases.

### Zymography

Zymography was performed to detect activity of proteases in gel using modified protocol followed by Bester et al [29]. The PPE (20 µl) was separated on denaturing polyacrylamide gel (12%) of 8.3*6.5 cm having SDS as a denaturant at 150V polymerised with and without 0.1% casein. After electrophoresis, polyacrylamide gel polymerised without casein was stained with Coomassie Brilliant Blue- R250 to estimate the molecular weight of the protease. The polyacrylamide gel containing 0.1% casein was soaked in 2.5% Triton X-100 for 1 hour followed by overnight incubation in 50 mM Tris buffer (pH 8) and then stained with Coomassie Brilliant blue-R250.

### Effect of physico-chemical parameters on protease production

Various culture parameters for production of alkaline protease by *Bacillus infantis* SKS1 were optimized. 1% casein (Sisco Laboratories Research, India) was added to 10 ml nutrient broth no. 2 (HiMedia, India) containing meat peptone, casein enzymic hydrolysate and sodium chloride at concentration of 4.3g/L, 4.3 g/L and 6.4g/L respectively in culture flask of 75 ml to observe the effect of temperature, pH, agitation, inoculum size and incubation time. To investigate effect of pH and temperature on production of alkaline protease, cells were grown in media having pH 6, 8, 10 and 12 incubated at temperatures 30ºC, 40 ºC and 50ºC. The optimized temperature and pH were kept constant for optimizing other parameters. The effect of agitation for alkaline protease production was studied at 50, 100 and 150 rpm in non-baffled flask. Keeping the above-mentioned optimized parameters constant, inoculum size varying from 1% to 90% and incubation time varying from 3 hours to 132 hours were optimized. Casein concentration for the production of alkaline proteases was optimized by adding casein (Sisco Laboratories Research, India) in nutrient broth at 0.5%, 1%, 2%, 3%, 4% and 5%, keeping other parameters constant.

Media containing yeast extract, sodium chloride and various carbon sources at 0.5% concentration was used to observe the effect of carbon sources on production of alkaline protease whereas media containing yeast extract and sodium chloride at 0.5% concentration without any carbon source was used as control. Media comprising starch, sodium chloride and various nitrogen sources at 0.5% concentration was used to observe the effect of nitrogen sources on production of alkaline protease whereas media containing starch and sodium chloride at 0.5% concentration without any nitrogen source was used as control. To examine the effect of metal salts on production of alkaline protease, different metal salts at 0.25% were added to production media keeping production media (nutrient broth containing casein) without metal salt as a control. Various carbon sources, nitrogen sources and metal salts as mentioned in the following table were optimized keeping other parameter constant (Table 4).

**Table 4:** Various carbon sources, nitrogen sources and metal salts used to optimize production.

Carbon sources (0.50%)	Nitrogen Sources (0.50%)		Metal salts (0.25%)
	Organic	Inorganic	
Glucose	Peptone	Sodium nitrate	Magnesium chloride
Fructose	Yeast extract	Ammonium phosphate	Potassium chloride
Xylose	Beef extract	Ammonium chloride	Manganese chloride
Dextrose	Malt extract	Ammonium sulphate	Calcium chloride
Glycerol	Tryptone	Ammonium oxalate	Cobaltous chloride
Starch	Casein		Lithium chloride
Mannitol	Gelatin		Cesium chloride
Sorbitol			Cadmium chloride
			Ferric chloride
			Sodium chloride
			Zinc sulphate
			Barium chloride

Various carbon sources (0.50%), nitrogen sources (0.50%) and metal salts (0.25%) were optimized for production of alkaline protease by *Bacillus infantis* SKS1.

### Statistical analysis

Results are represented as mean ± standard error of three replicates. Statistical analysis was done using Assistat software [30] by one-way analysis of variance (ANOVA). Tukey’s test (Tukey method) is a multiple comparison test that is applicable for comparison of more than two means [31]. This statistical test is used to determine the individual means that is significantly different from a set of means. It is used after an Analysis of Variance, which shows the existence of significant differences.

## Results

### Morphological characterization of strain SKS1

*Bacillus infantis* SKS1 isolated from garden soil was capable of producing alkaline protease as the zone of clearance was observed around colonies on skim milk agar plate (pH 10) stained with congo red. The shape of colony of this rod shaped Gram-positive bacteria was observed as entire, translucent, smooth and beige on nutrient clerigel plates under stereomicroscope. This culture was submitted to MCC, Pune (India) with accession number MCC 3035.

### Biochemical characterization of strain SKS1

*Bacillus infantis* SKS1 was biochemically characterized by examining the ability for carbohydrate utilization, enzyme hydrolytic activities and production of indole, stable acids and acetylmethylcarbinol. The capability of the strain for utilization of carbohydrates was analysed using HiCarbo kit (Table 5). According to our results using HiCarbo kit, *Bacillus sp*. SKS1 was found to utilize monosaccharides including fructose, dextrose and xylose while unable to utilize arabinose, mannose, galactose, rhamnose and sorbose. The isolate was unable to utilize monosaccharide derivatives except malonate, salicin and esculin available in HiCarbo kit. It was unable to utilize trisaccharides while capable of utilizing maltose and trehalose amongst other disaccharides and sugar alcohols such as sorbitol, mannitol and glycerol as per HiCarbo kit whereas *Bacillus infantis* SMC 4352–1 was incapable of utilizing glycerol as reported by Ko et al. [32]. The strain was capable of producing amylase and lipase while incapable of producing indole, lysine decarboxylase and acetylmethylcarbinol (Table 6).

**Table 5:** Carbohydrate utilization tests of strain SKS1.

Positive result	Negative result
Xylose utilization	Lactose utilization
Maltose utilization	Galactose utilization
Fructose utilization	Raffinose utilization
Dextrose utilization	Melibiose utilization
Trehalose utilization	Sucrose utilization
Inulin utilization	L-Arabinose utilization
Glycerol utilization	Mannose utilization
Salicin utilization	Sodium gluconate utilization
Malonate utilization	Dulcitol utilization
Sorbitol utilization	Inositol utilization
Esculin hydrolysis	Adonitol utilization
Mannitol utilization	Arabitol utilization
	Erythritol utilization
	ɑ-Methyl-D-glucoside utilization
	Rhamnose utilization
	Cellobiose utilization
	Melizitose utilization
	ɑ-Methyl-D-mannoside utilization
	Xylitol utilization
	ONPG test
	D-Arabinose utilization
	Citrate utilization
	Sorbose utilization

The ability of *Bacillus sp*. SKS1 to utilize various carbohydrates was analysed using HiCarbo kit.

**Table 6:** Biochemical characterization of strain SKS1.

Biochemical test	Interpretation	Result
Spirit blue agar test	Production of lipase	Positive
Starch agar test	Production of amylase	Positive
Lysine decarboxylase test	Production of lysine decarboxylase	Negative
Tryptone water test	Production of indole	Negative
Methyl red test	Production of stable acids by fermentation of glucose	Positive
Vogues Proskauer test	Production of acetylmethylcarbinol	Negative

Various biochemical tests were performed for characterization of strain SKS1 possessing various hydrolytic activities.

Antibiotic susceptibility tests were performed using disc diffusion method (Table 7). The strain was resistant to cefepime while it was sensitive to other β- lactam antibiotics that are responsible for inhibition of cell wall synthesis such as ceftriaxone, penicillin G, augmentin and glycopeptide antibiotics such as vancomycin [33]. The isolate was sensitive to antibiotics inhibiting protein synthesis such as doxycycline (tetracycline), quinupristine/dalfopristin (streptogramin), erythromycin (macrolide), fusidic acid, chloramphenicol and aminoglycosides. The isolate was also sensitive to quinolone antibiotics such as levofloxacin, sparfloxacin and ciprofloxacin that are responsible for inhibition of bacterial topoisomerase IV and DNA gyrase, the enzymes, which are required for DNA replication, transcription, repair and recombination [33]. Sulphonamide antibiotics such as co-trimoxazole responsible for blockage of synthesis of nucleic acids and essential proteins were also effective against this isolate.

**Table 7:** Antibiotic susceptibility test of strain SKS1.

Antibiotic	Result
Doxycycline (30 μg)	Sensitive
Levefloxacin (5 μg)	Sensitive
Ceftriaxone (30 μg)	Sensitive
Quinupristine/dalfopristin (15/15 μg)	Sensitive
Co-trimoxazole (25 μg)	Sensitive
Amikacin (30 μg)	Sensitive
Sparfloxacin (5 μg)	Sensitive
Penicillin G (10 units)	Sensitive
Gentamycin (10 μg)	Sensitive
Augmentin (30 μg)	Sensitive
Ciprofloxacin (5 μg)	Sensitive
Erythromycin (10 μg)	Sensitive
Fusidic acid (10 μg)	Sensitive
Chloramphenicol (30 μg)	Sensitive
Vancomycin (30 μg)	Sensitive
Cefepime (30 μg)	**Resistant**

Antibiotic sensitivity test was performed which shows that strain SKS1 was resistant against cefepime while it was sensitive to other antibiotics.

### Molecular characterization of strain SKS1

**Fig 1:**
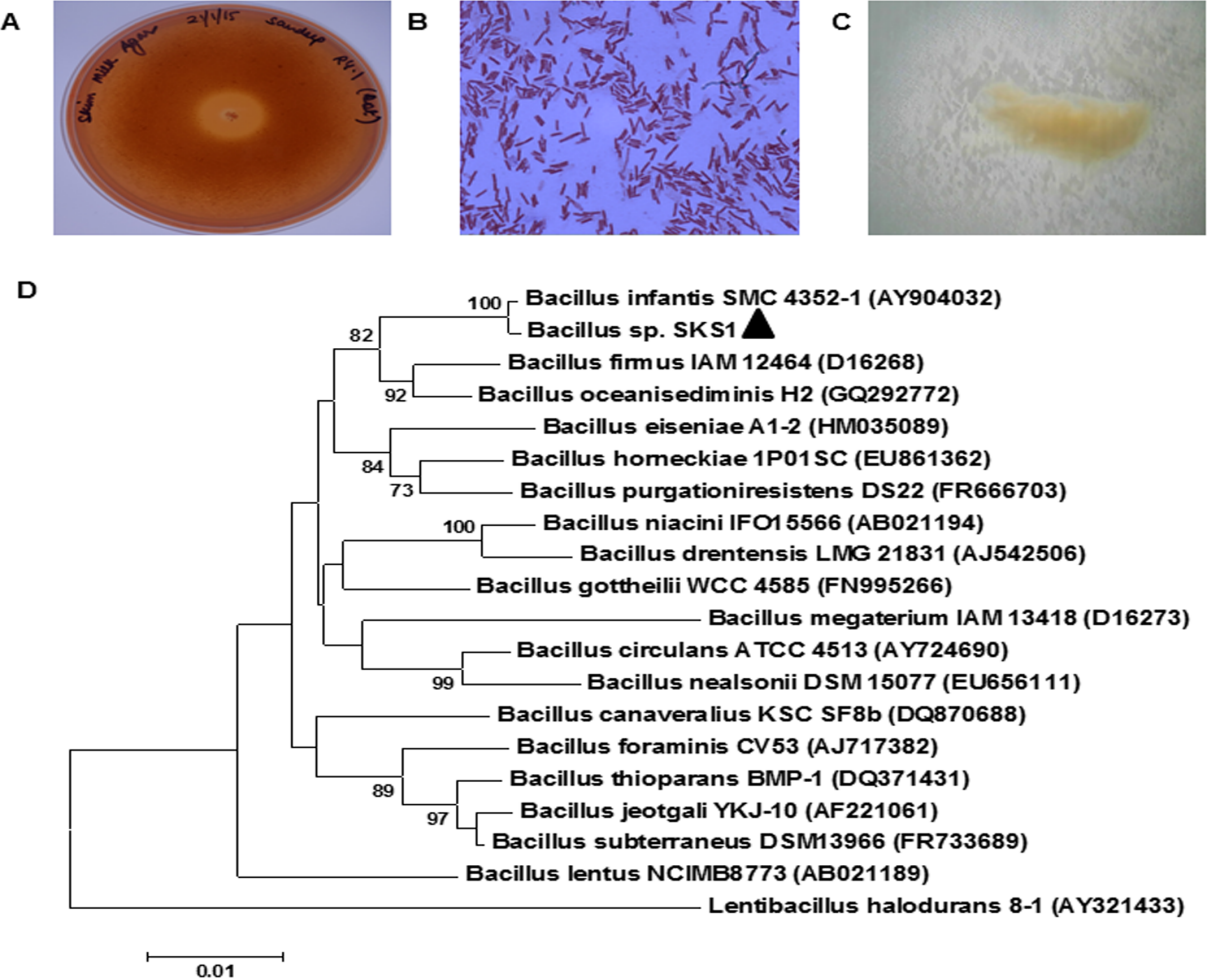
Morphological characterization of *Bacillus sp*. SKS1. **(A)** Clear zone surrounding colony of *Bacillus sp*. SKS1 showing protease activity on skim milk agar stained with Congo red. **(B)** Gram staining of *Bacillus sp*. SKS1 showing Gram-positive rods. **(C)** Entire, translucent, beige and smooth colony of *Bacillus sp*. SKS1. **(D)** The phylogenetic tree based on neighbour-joining method of *Bacillus sp*. SKS1 and its homologs based on 16S rDNA sequences using Mega 6 software. Bootstrap values based on 1000 replicates are shown at branch nodes. The sequence of 16S rDNA of *Lentibacillus halodurans* 8–1 was used as an outgroup.

Molecular characterization of bacteria was performed using sequence of 16S rDNA, a conserved gene generally used for species identification. PCR amplification of 16S rDNA gene was performed using universal primers and genomic DNA as template. The sequence of 16S rDNA containing 1420 bp was deposited to GenBank having accession number KR092197 (http://www.ncbi.nlm.nih.gov/nuccore/KR092197). This sequence was used to search RDP-II, EzTaxon and NCBI database using BLASTn. BLASTn results against NCBI database showed 99% sequence identity to a sequence of *Bacillus infantis* strain FM 34 and *Bacillus sp*. Beige. Result of RDP depicts maximum similarity to the phylum Firmicutes, class Bacilli, order Bacillales, family Bacillaceae 1 and genus *Bacillus*. EzTaxon result shows 99.85% sequence identity to *Bacillus oceanisediminis* H2 (T). *Bacillus sp*. SKS1 is present in a cluster with *Bacillus infantis* SMC 4352–1 in the evolutionary tree formed using neighbor-joining method supported by a bootstrap value of 1000 (Fig 1D).

### Characterization of alkaline protease

Ammonium sulphate precipitation was used for partial purification of protease produced from *Bacillus infantis* SKS1 (Table 8). The total activity of culture supernatant and precipitated enzyme was 375 U and 328.5 U respectively. The specific activity of precipitated enzyme was increased to 21.27 U/mg of total protein having a yield of 87.6% with 2.68 purification fold. The enzyme precipitated with ammonium sulphate followed by dialysis was used to study its characteristics.

**Table 8:** Purification of enzyme produced by *Bacillus infantis* SKS1.

Purification step	Total activity (U)	Total protein (mg)	Specific activity (U/mg)	Yield (%)	Purification fold
Culture supernatant	375	47.27	7.93	100	1
Ammonium sulphate precipitation	328.5	15.44	21.27	87.6	2.68

Partial purification of enzyme produced by *Bacillus infantis* SKS1 was achieved using ammonium sulphate precipitation method.

**Fig 2:**
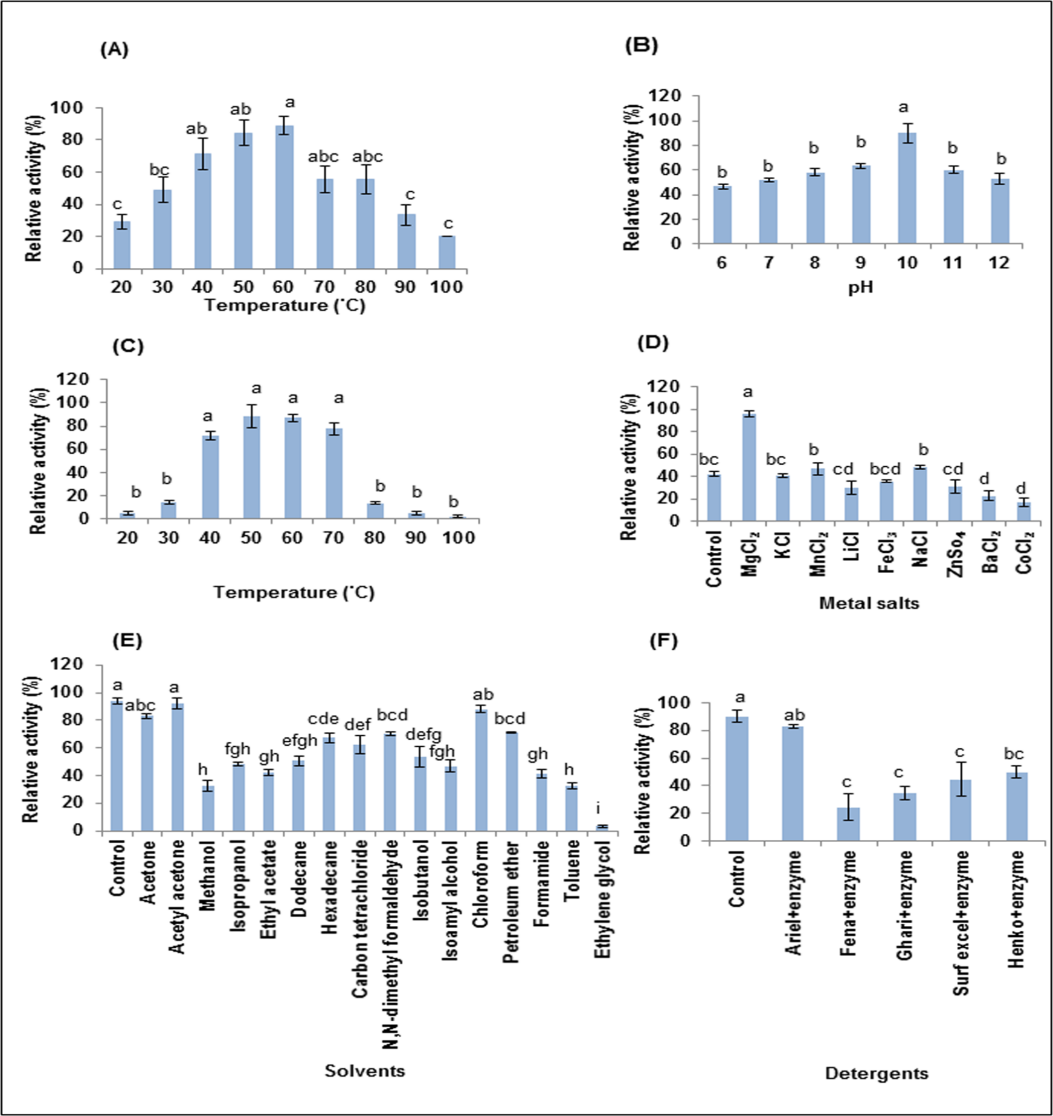
Characterization of alkaline protease. Effect of **(A)** preincubation at various temperatures (F = 11.345, p<0.0001), **(B)** pH (F = 13.307, p<0.0001), **(C)** temperature (F = 90.359, p<0.0001), **(D)** metal salts (F = 41.762, p<0.0001), **(E)** solvents (F = 48.748, p<0.0001), **(F)** laundry detergents (F = 13.571, p<0.0001) on activity of alkaline protease. Columns and error bars above column represent Mean±SEM respectively. Different letters above the bar are statistically different whereas same letter do not differ between them as determined by Tukey’s test.

Our study shows that partially purified extract (PPE) was stable up to 60°C (F = 11.345, p<0.0001) when it was pre-incubated at different temperatures ranging from 20°C to 100°C (Fig 2A). Alkaline proteases are usually stable at 50°C-70°C with an exception of *Bacillus sp*. B18 having stability at a temperature of 85°C [34]. In our study, partially purified extract (PPE) showed maximum activity at pH 10 (F = 13.307, p<0.0001) when reaction mixture was incubated at pH ranging from 6 to 12 (Fig 2B).

The reaction mixture was incubated at different temperatures ranging from 20°C to 100°C to study effect of temperature on enzyme activity (Fig 2C). Our results show that the partially purified extract (PPE) was active at wide range of temperatures ranging from 40°C to 70°C (Fig 2C) and showed its maximum activity at 50°C (F = 90.539, p<0.0001). Large amount of detergent is needed during washing of clothes in hard water as presence of Mg^2+^ and Ca^2+^ interfere with cleaning of clothes. Our results show that Mg^2+^ significantly enhanced the enzyme activity (F = 41.762, p<0.0001) suggesting suitability of partially purified extract (PPE) containing proteases in laundry detergents for washing in hard water (Fig 2D).

Enzymatic synthesis of peptides require proteases that are stable in organic solvents [35]. Our study reveals that PPE of *Bacillus infantis* SKS1 retained enzyme activity in presence of acetyl acetone (F = 48.748, p<0.0001) emphasizing its usage in enzymatic synthesis of peptides in presence of solvents (Fig 2E). The activity of PPE in presence of laundry detergents (solid) was also determined by incubating the reaction mixture with detergents of different brands for an hour. The PPE retained 82.64%, 24.31%, 34.77%, 44.44% and 50% relative activity in presence of Ariel, Fena, Ghari, Surf excel and Henko respectively (F = 13.571, p<0.0001) (Fig 2F).

### Zymography

**Fig 3:**
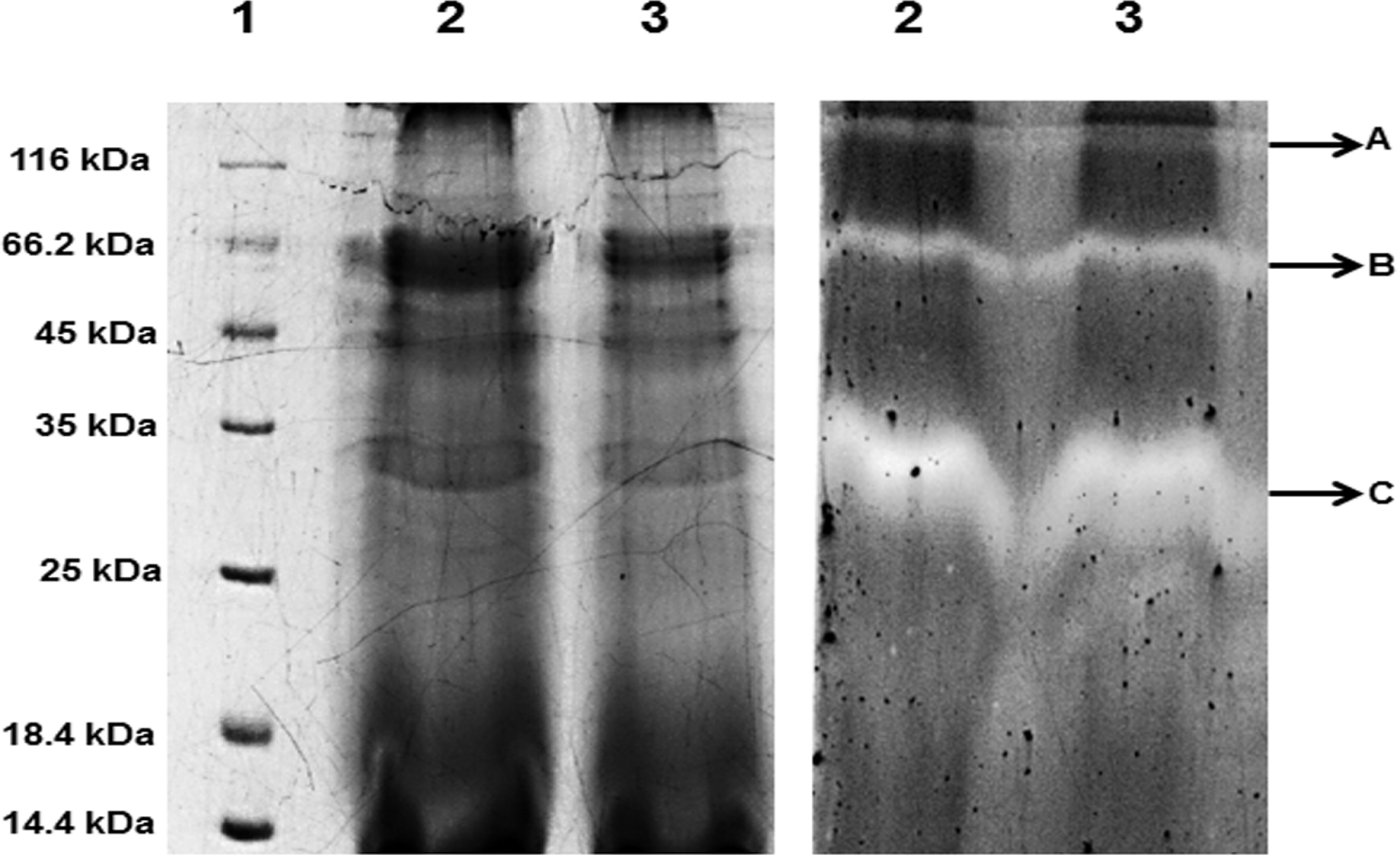
SDS-PAGE zymogram analysis of alkaline protease from *Bacillus sp*. SKS1. 12% SDS-PAGE (left) and casein zymogram (right) of ammonium sulphate precipitate of culture supernatant obtained from *Bacillus sp*. SKS1 showing three protease bands of approximately (A) 120 kDa, (B) 66 kDa and (C) 35 kDa. (Lane 1: unstained marker, lane 2: ammonium sulphate precipitated protease, lane 3: ammonium sulphate precipitated protease heated at 60°C for an hour).

Casein zymography is a SDS-PAGE based electrophoretic method used to detect in gel protease activity using casein as a substrate [29]. The partially purified extract exhibiting protease activity was separated on SDS-PAGE polymerized with casein and soaked in Triton X-100 followed by incubation in 50 mM Tris buffer (pH 8) overnight. The Coomassie Brilliant Blue-R250 stained gels showed three clear zones in casein zymogram due to casein hydrolytic activities indicating the presence of three proteases of molecular weight of approximately 120 kDa, 66 kDa and 35 kDa. Casein zymogram using partially purified extract heated at 60°C was able to show all three clear zones showing that proteases were stable at 60°C (Fig 3).

### Effect of physico-chemical parameters on alkaline protease production

**Fig 4:**
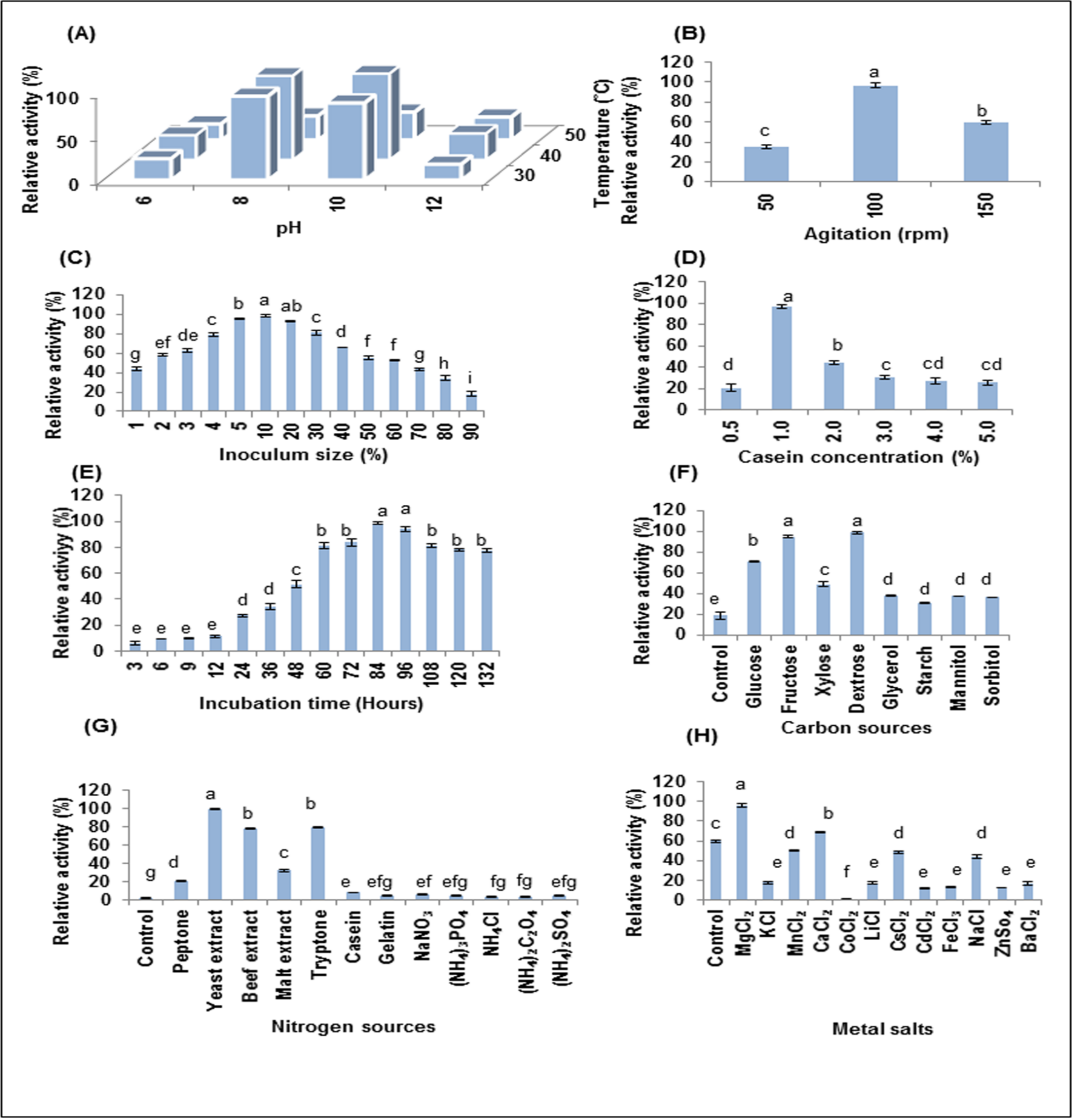
Optimization of physico-chemical parameters for production of alkaline protease. Effect of **(A)** temperature and pH (F = 120.39, p<0.01), **(B)** agitation (F = 359.376, p<0.0001), **(C)** inoculum size (F = 304.37, p<0.0001), **(D)** casein concentration (F = 120.898, p<0.0001), **(E)** incubation time (F = 473.109, p<0.0001), **(F)** carbon sources (F = 300.684, p<0.0001), **(G)** nitrogen sources (F = 2340.972, p<0.0001) and **(H)** metal salts (F = 469.588, p<0.0001) on production of alkaline protease. Columns and error bars above column represent Mean ± SEM respectively. Different letters above the bar are statistically different whereas same letter do not differ between them as determined by Tukey’s test.

Incubation temperature and pH have profound effect on cell growth and production of extracellular enzyme. Our study shows that the optimum production of enzymes was observed at 40°C and pH 10 (Fig 4A). The increase in temperature beyond 40°C and pH beyond 10 decreased the production of proteases. Keeping optimized pH and temperature constant, the maximum production of alkaline proteases was observed at 100 rpm (F = 359.376, p<0.0001) whereas 51.07% and 28.19% of maximum production was observed at 50 rpm and 150 rpm respectively (Fig 4B). Maximum production of alkaline proteases was observed using 10% inoculum and 1% casein in production media (Fig 4C and 4D). Since microbes secrete extracellular enzymes during specific phase of their growth period, production was observed till 132 hours keeping other optimized parameters constant. The optimum time required for production by *Bacillus infantis* SKS1 was 84 hours (Fig 4E) after which a slight decline in production was observed may be due to decomposition of enzyme (F = 473.109, p<0.0001).

According to previous reports, it has been observed that carbon sources have significant effects on protease production [36]. In case of *Bacillus infantis* SKS1, results show that dextrose and fructose were the best carbon sources for maximum production (F = 300.684, p<0.0001). The enzyme production was increased to 98.60% and 94.55% using dextrose and fructose respectively as a sole carbon source while glucose and xylose increased the enzyme production to 70.98% and 48.92% respectively (Fig 4F). The use of glycerol, starch, mannitol and sorbitol as a sole carbon source in media increased the production of alkaline protease to 37.70%, 30.97%, 37.55% and 36.41% respectively. Media containing 0.5% yeast and 0.5% sodium chloride without carbon source was used as control. Various nitrogen sources enriched in peptides and amino acids affect the production of alkaline protease by the microbe (Fig 4G). The maximum production was observed using yeast extract as a nitrogen source (F = 2340.972, p<0.0001) whereas media containing 0.5% starch and 0.5% sodium chloride without any nitrogen source was used as control. Other organic nitrogen sources such as peptone, malt extract and casein increased the protease production to 21.25%, 32.72% and 8.39% respectively while very slight increase in production was observed using inorganic sources of nitrogen. Metal salts are one of the other major factors that influence production of protease (Fig 4H). On addition of 0.25% metal salts like calcium chloride and magnesium chloride in production media, there was a significant increase in protease production (F = 469.588, p<0.0001). Cesium chloride, manganese chloride and sodium chloride slightly decreased the protease production to 48.44%, 50.61% and 44.19% of the maximum production respectively and cobaltous chloride was observed to decline the production to 0.85%.

## Discussion

Microbes are important source for production of alkaline proteases, as bacterial proteases possess most of the features required for industrial applications [6]. This study deals with isolation, identification and characterization of a bacteria isolate from garden soil of north India that produces alkaline proteases and has 99% sequence identity with *Bacillus infantis*. This is the first report of production and characterization of alkaline protease produced by any strain of *Bacillus infantis* as per best of our knowledge. Biochemical characteristics of *Bacillus infantis* SKS1 showed that it was capable of utilizing fructose, dextrose and trehalose. The utilization of inexpensive substrates such as molasses (source of fructose and dextrose) and corn steep liquor (source of fructose and trehalose) for production of enzymes may reduce the cost of fermentation as 30–40% production cost depends on the media components [37].

This study reports characterization of PPE of *Bacillus infantis* SKS1 obtained through ammonium sulphate precipitation. The PPE of *Bacillus sp*. SKS1 was active at pH 10 and wide range of temperatures (40°C to 70°C) suggesting its application in industry demanding moderate heat and alkaline conditions. Some cations are needed by detergent-compatible enzymes to protect their active site against thermal denaturation during washing at higher temperatures [36]. Our results show that the PPE of *Bacillus infantis* SKS1 showed increase in activity in presence of Mg^2+^ whereas protease activity was decreased in presence of Ba^2+^, Co^2+^ and Zn^2+^. As per previous reports, cations such as Ca^2+^, Mg^2+^, Mn^2+^ and Cu^2+^ enhance the enzyme activity while metal ions such as Hg^+^ and Zn^2+^ are responsible to inhibit the proteolytic activity [36]. The PPE was quite stable in solvent such as acetyl acetone as it retained 83% activity suggesting its application in enzymatic synthesis of peptides in presence of organic solvents [35]. For an enzyme to be a good candidate for detergent preparations, it should be compatible with chemicals and ingredients present in the laundry detergents. Several reports suggest that enzyme produced by a strain is not compatible with all the available laundry detergents in the market. Vaishali Choudhary reported that *Aspergillus versicolor* protease retained maximum activity of 76% with Ghadi detergent and the least with Tide laundry detergent whereas protease produced by *Bacillus cereus* retained more than 80% activity in presence of laundry detergents [38, 39]. Bhosale et al reported that alkaline protease from *Conidiobolus coronatus* (NCL 86.8.20) was most compatible with Revel followed by Ariel and Wheel [40]. The PPE retained 82.04% activity on incubation with laundry detergent named Ariel making it a good candidate for wash.

The protease produced by *Bacillus pumilis* MK6-5 [41] was active at 50°C-55°C (pH 11) whereas *Bacillus licheniformis* [42] and *Bacillus firmus* 7728 [43] produced proteases which are active at 37°C (pH 8.5) and 40°C (pH 9) respectively. Our results show that the PPE of *Bacillus infantis* SKS1 showed its maximum activity at 50°C (pH 10) as it was thermostable under alkaline conditions (Table 9).

**Table 9:** Comparison of microorganisms capable of producing alkaline proteases.

Microbe	Optimum pH for activity	Optimum temperature for activity	Optimum pH for activity	Optimum temperature for production	Optimum agitation rate for production	References
*Alcaligenes faecalis*	9	55°C	8	30°C	200 rpm	[44]
*Bacillus sp*. JB99	11	70°C	10	55°C	180 rpm	[45]
*Bacillus sp*. SSR1	10	40°C	10	40°C	150 rpm	[46]
*Bacillus brevis* MTCC B0016	10.5	37°C	10.5	37°C	200 rpm	[47]
*Bacillus mojavensis*	10.5	60°C	7	50°C	200–250 rpm	[48]
*Oligotropha carboxydovo-rans* DSM1227	9	60°C; 50°C	-	-	-	[49]
*Bacillus pumilis* MK6-5	11.5	50–55°C	9.6	35°C	250 rpm	[41]
*Bacillus subtilis* RJAS 19	9.5	65°C	9	50°C	-	[50]
*Bacillus licheniformis* MZK-03	8.5	37°C	5–12	37°C	200 rpm	[42]
*Bacillus firmus* 7728	9	40°C	9	40°C	120 rpm	[43]
*Bacillus infantis* SKS1	10	50°C	10	40°C	100 rpm	

Production parameters and characteristics of alkaline proteases produced by various microorganisms were compared.

Commercial application of an enzyme depends upon its characteristics, cost and its availability. Large scale production of industrial enzymes require fermenter where various parameters like media components, temperature, pH, inoculums size, agitation etc. have significant impact on product production and cost of its operation Incubation temperature has a role in energy metabolism, translational synthesis of proteins and it affects physical properties of cell membrane [12]. During fermentation, temperature increases with time leading to huge amount of energy requirement for cooling of fermenter. Thus, bacteria growing at higher temperature would be more suitable for fermentation leading to reduction in energy consumption and cost. Our results show that the optimum incubation temperature for production of enzymes in case of *Bacillus infantis* SKS1 was 40°C, which is similar to other microbes like *Bacillus cereus*, *Bacillus subtilis* PE-11 and *Bacillus licheniformis* KBDL4 [36]. Agitation is another important factor that plays an important role in transfer rate of nutrients, oxygen transfer, cell aggregate dispersion and increased aerobic metabolism of microbe [12]. During fermentation process, energy consumed by stirrer and aerator influence the overall cost of the enzyme production [51]. Our results show that the maximum production of enzyme was observed at low agitation rate (i.e. 100 rpm) in case of SKS1 whereas high agitation rates (120–250 rpm) are required for microorganisms such as *Bacillus pumilis*, *Bacillus licheniformis* and *Bacillus firmus* that are used for commercial production of alkaline proteases (Table 9).

Media components such as carbon sources have been important for production of alkaline proteases as they play role in biosynthesis and energy generation. It has been reported that xylose was the best carbon source for production of proteases from *Bacillus licheniformis* (TD4) [52] while glucose was the best carbon source for *Bacillus subtilis* NS [53]. In this study, dextrose and fructose were the best carbon sources for maximum production of alkaline protease using *Bacillus infantis* SKS1. Inexpensive carbon sources such as beet molasses and corn steep liquor, which are a rich source of fructose and dextrose respectively, can be used for large-scale production of proteases in industries. Microbes metabolize nitrogen sources to produce peptides and amino acids for enzyme production [36]. Organic nitrogen sources result in better production of alkaline proteases in case of SKS1 as compared to inorganic nitrogen sources. This suggests the use of other organic inexpensive industrial by-products such as wheat bran, peanut meal, rice bran, soybean meal etc. in solid-state fermentation as high volumetric and concentrated product can be recovered easily in comparison to submerged fermentation [54]. Moreover, the solid-state fermentation is ecofriendly due to the usage of agro industrial residues as substrates and low waste water output [54]. Thus, solid-state fermentation can be explored for the production of enzymes by *Bacillus infantis* SKS1.

## Conclusion

*Bacillus infantis* SKS1 isolated from garden soil of northern region of India are Gram-positive rods. Molecular characterization performed using 16S rDNA sequencing reveals its 99% sequence identity with *Bacillus infantis* strain FM 34 and *Bacillus sp*. Beige. The PPE of this isolate was active over wide range of temperatures under alkaline conditions. The thermostability and retention of protease activity in presence of acetyl acetone and local laundry detergent may result its utilization in industrial processes. Various parameters for production of alkaline proteases from *Bacillus infantis* SKS1 were optimized so that large-scale production of enzyme can be achieved in future.

## Abbreviations

$: US dollar
°C: centigrade
rpm: revolutions per minute
ml: millilitres
N: normal
nm: nanometres
g/L: grams per litre
μg: micrograms
mM: millimolar
mg: milligrams
MCC: Microbial Culture Collection
ONPG: ortho-Nitrophenyl-β-galactoside
PCR: polymerase: chain reaction
bp: base pairs
NCBI: National Center for Biotechnology Information
BLAST: Basic Local Alignment Search Tool
RDP-II: Ribosomal Database Project-II
SEM: standard error mean
SDS-PAGE: sodium dodecyl sulfate polyacrylamide gel electrophoresis
ANOVA: analysis of variance
rDNA: ribosomal deoxyribonucleic acid
PPE: Partially purified extract
